# Investigation of Each Society for Fertility Preservation in Asia

**DOI:** 10.3389/fendo.2019.00151

**Published:** 2019-03-14

**Authors:** Achmad Kemal Harzif, Victor Prana Andika Santawi, Mila Maidarti, Budi Wiweko

**Affiliations:** Faculty of Medicine, Universitas Indonesia, Jakarta, Indonesia

**Keywords:** fertility preservation, cancer, Asian countries, reproductive technology, cancer therapy

## Abstract

Fertility preservation (FP) remains a future direction for reproductive medicine. FP development is needed to keep up with advancements in other areas of medicine, such as cancer research that has successfully prolonged patients' survival. The demand for optimum FP is sought by both patients and practitioners alike. The American Society of Clinical Oncology has published and updated several FP guidelines. However, these guidelines need to be optimized for each country due to the nature of FP that interacts with the local policy, social decorum, and economic factors. Furthermore, the availability and success rate for each procedure might differ since there is a requirement for advanced and innovative technologies involved in FP. These guidelines should ideally be supported by the FP society to overcome unique barriers that may arise in each country. Here we try to illustrate the most recent update on the condition of FP practice in several countries in Asia. This hopefully will encourage future FP development and might become a useful reference for other countries, especially in Asia.

## Introduction

Cancer remains highly prevalent despite discoveries of its risk factors. In 2012, there were 14.1 million new cases and around 8.2 million deaths were caused by cancer. In Indonesia, the prevalence of cancer of all ages in 2013 was 1.4‰ or estimated at 347,792 people (1). Breast cancer, lymphoma, skin cancer (excluding basal and squamous types), and leukemia are the most prevalent cancers in young adults aged 20–44 years (1). The adverse outcome of infertility due to premature gonadal failure is prominent, despite the overall 5-year relative survival rate improvement to 82.7% for individuals younger than 45 years (2). In 2017, an estimated 15,270 children and adolescents ages 0–19 were diagnosed with cancer (2).

Patients with cancer can now live longer owing to advances in diagnosis and treatment. Their lives, however, are handicapped by long-term effects of cancer and its treatments, in terms of being psychological, economic, social, sexual, and biological. The commonly prescribed cancer treatment can negatively impact major female reproductive systems and may lead to the loss of reproductive organs, premature ovarian failure, or an inability to produce mature eggs (3). Women also face the risk of immediate or premature menopause (4). In addition to the biological consequences, loss of fertility can be devastating for younger adults with respect to its severe and long-lasting emotional impact (3).

Fertility preservation (FP) is a developing field that envelops an assortment of fertility treatments for patients envisioning restorative treatment that could influence future conceptive results (5). The first widely-known guideline produced was by American Society of Clinical Oncology (ASCO) in 2006 for patients facing cancer therapy (6). FP is often linked with cancer treatment, however, it has also been applied to patients who require fertility affecting treatments, such as lupus, glomerulonephritis, and myelodysplasia. Furthermore, FP can also be applied for adolescent females with Turner mosaicism or other conditions that cause premature ovarian failure. The 2006 ASCO Recommendations on FP in Cancer Patients advises that all oncologists should address potential treatment-associated infertility with patients of childbearing age. This is based on the fact that cancer survivors believed that they will be a better parent upon surviving cancer which drove their intention toward pregnancy (7).

## Fertility Preservation Options

A recent ASCO guideline published in 2018 did not differ significantly from the 2013 guideline. Notwithstanding, the 2013 ASCO is more focused on promoting a holistic approach to include all providers to discuss FP to cancer patients (8). More importantly, it emphasizes the use of Gonadotropin-Releasing Hormones agonist (GnRHa) as an option for FP. In contrast, ASCO 2018 indicates that GnRHa should not be used to replace other means of more reliable FP options whenever possible despite a lower likelihood of chemotherapy-induced ovarian failure is noted with the use of GnRHa. ASCO 2018 added recommendations that all patients in reproductive age with cancer are routinely counseled about the effect of cancer treatment on fertility and all options available.

## Ethical Consideration

Several arguments exist to support the use of fertility preservation. One would argue that everyone has the right to reproduce as a basic human right (4). Several studies concluded that given the right support system and counseling, patients undergoing fertility preservation procedures are satisfied (9–11). Fertility preservation has given hope to some patients and give a stronger reason to live (12). Without proper counseling, these benefits may instead cause anxiety and depression among the cancer patients (13, 14). Consideration should also be taken when the right to reproduce is outweighed by others' right. This concerns the welfare of the children born with a higher chance of losing the parent in addition to the possibility of inheriting the disease (4).

Another argument would be a non-maleficence aspect of healthcare providers, as it is our duty to do no harm, or at least reverse the harm done by treatment whenever possible (15). This can also be debated with the possibility of delay in curative treatment from the fertility preservation procedures. Despite the expected longer delay in the cancer treatment, mortality, and recurrence rate does not differ between women who underwent fertility preservation and not (16).

Although ethical aspects often change over the course of history, it can be inferred from recent studies that cancer survival rate is higher than ever. Thus, it is ethical to provide FP options when it is accompanied with appropriate counseling.

## Asian Society for Fertility Preservation

The Asian Society for Fertility Preservation (ASFP) already established and there are many countries that have joined the society, which are Japan, Hong Kong, India, Singapore, Korea, Taiwan, Thailand, Indonesia, Vietnam, Philippines, China, and Pakistan. ASFP is the first initiative of experts from all over Asia to promote FP science and practice. Being the first, ASFP sparks countries in Asia to establish their own FP society. ASFP's mission is to raise awareness of FP among medical professionals and the public, to improve technical skills, and to keep health practitioners informed about the latest developments in the field and in a research setting (17).

## Japan

Japan Society for Fertility Preservation (JSFP) is a non-profit organization found in 2012 aiming to appropriately organize, implement, and understand the healthcare system for oncofertility therapy. While ASCO is readily accessible, it is not necessarily applicable to Japanese Cancer patients (18). They also serve to provide patients with issues regarding oncofertility treatments among healthcare professionals from multiple specialties. The society states that “treatment of the patient's malignancy must receive the highest priority,” thus FP should be completed within a limited period. It then becomes the treating physicians' responsibility to inform the patient whether to abandon FP and suspending anticancer treatment unnecessarily (19). Written in a native language, JSFP website provides information for patients and healthcare professionals.

Japan has published several guidelines from Japanese Society for Reproductive Medicine (JSRM) and Japanese Society of Obstetrics and Gynecology (JSOG). These guidelines, however, are not comprehensive enough to address FP for various cancer types (18). In 2017, the guideline on FP for young cancer patients has been published by the Japan Society of Clinical Oncology that can be applied by healthcare providers. Options available for female patients in Japan are oocyte, embryo, and ovarian tissue cryopreservation. Oocyte cryopreservation has been the first option as a method for FP in Japan. However, in the case when the delay for cancer treatment is not tolerable or increased estradiol due to ovarian stimulation may cause adverse effects on cancer, ovarian tissue cryopreservation is preferred.

As a safeguard, all frozen oocyte or embryo should be reported by the physician to JSOG (20). Oocyte cryopreservation is considered as a medical practice to counter the adverse effects of cancer therapy and patients should be informed of this option regardless of their desire for childbirth at the time. Patients should also be informed about the procedures impacts on the disease prognosis, the possibility and safety of pregnancy in the future, so that they can make their own independent decision (18).

Among patients who are eligible for oocyte cryopreservation, ovarian tissue cryopreservation can still be offered. The only contraindication is when the cancer cells might be present in the ovarian tissue. The method for ovarian tissue cryopreservation in Japan favors the use of closed method vitrification, which is different from the recommended slow-freezing technique by ASRM 2013 (21). They have conducted a systematic review and meta-analysis and concluded that the vitrification technique is superior in terms of DNA strand breaks and stromal cells preservations (22). This technique is expected to be used as standard method of ovarian tissue cryopreservation in the near future. After the tissue cryopreservation, auto-transplantation can be done with a successful birth rate of 25% (23). Alternatively, the immature oocytes can be collected from antral follicles and cultured for further growth in a specific medium for oocytes *in vitro* maturation.

FP option for male patients is restricted to sperm cryopreservation. When facing difficulty in extracting sperm, Penile Vibratory Stimulator, Electroejaculation under general anesthesia, or Microsurgical Testicular Sperm Extraction (TESE) can also be an option. Testicular tissue cryopreservation remains restricted to experimental research as it is not possible to mature spermatogonial stem cells *in vitro*. However, since it is the only option for pre-pubertal boys it can still be done with the hope that auto-transplantation may resume sperm production (18).

Japanese Government also enforced Law regarding FP. With regards to cost, Japan has not established coverage for the patients. Insurance also does not cover cryopreservation procedures thus patients are asked to fund the procedures themselves including consultation fee. One survey around the globe reports Japan has one of the highest cost of FP procedures with the cost ranging from 150 to 8,000 USD$ (24).

## Republic of Korea

In 2013, Korean Society for Fertility Preservation (KSFP) was established to ease teamwork between medical doctors and researchers specializing in reproductive medicine and oncology. The aim of KSFP is to help the patients who undergo treatments that may affect fertility. The society also has established standard protocols and policies including referral system for optimum FP application through an annual conference and postgraduate courses. As a result, Korea has a well-established network for their hospitals including the regional ones. This ensures the availability of high-quality FP treatments in each institution. As a global partner of Oncofertility Consortium, together with JSFP, they share information and experience for improvement of oncofertility research and clinical programs in Asia (25). KSFP does not limit the application of FP to oncologic patients, instead all patients facing treatment-related infertility such as lupus, rheumatoid arthritis, or Crohn disease, and those planning to undergo bone marrow or stem cell transplantation for hematologic diseases are also candidates (26).

KSFP guidelines promote a multidisciplinary team approach, involving physicians, nurses, mental health professionals, office staff, and laboratory personnel. This is done to understand patients' unique situation and develop the flow system to build successful FP program (26). Several guidelines are also published regarding FP for breast cancer (27), hematologic malignancies (28), and gynecologic malignancies (29). These guidelines provide considerations between FP techniques for specific cases. In addition to similar recommendations with 2018 ASCO guideline, gonadal shielding and ovarian transposition is encouraged for gynecological malignancies (29).

KSFP acknowledges that the time pressure for cancer treatment causes patients having difficulties in processing the information regarding FP. Therefore, oral, printed materials, and web-based resources need to be prepared (30), as well as encouraging patients to ask and additional FP consultations with fertility specialist (31) to ensure patients understand the risk of the chosen FP and cancer treatments. It is also stated in the guideline that despite clinical judgement may be used whether FP techniques are appropriate, early and prompt referral should be made to minimize time delay to begin the cancer treatment (26).

Despite the effort, a review stated that Korea faces barriers for FP in terms of issues with referrals, a financial burden for patients, and an inability to secure funding for research. Since there is no insurance coverage, patients must pay roughly $US 2,000–3,000. For ovarian tissue cryopreservation, only operation costs may be partially covered by insurance, which results to the same costs for ovarian tissue cryopreservation and oocyte or embryo cryopreservation (24).

## India

Fertility Preservation Society of India (FPSI) is the first organization in India to promote the science and practice of fertility preservation mainly for cancer treatment but also for some diseases that can cause premature infertility (Premature Ovarian Failure, Autoimmune diseases, and Fragile X Syndrome). They vision to promote science and practice of FP in India and active interaction between medical specialties to accomplish patient's reproductive health expectations and quality of life regarding FP. They claim that all patients should receive reproductive information, referrals, and decision making supports about FP from the healthcare professionals (32).

India offers embryo cryopreservation, oocyte cryopreservation, gonadal shielding, and ovarian transposition surgery as standard FP options for post-pubertal to pre-menopausal female patients aged 15–45 years old. The ovarian transposition option, however, is reserved for patients who are not eligible for embryo or oocyte cryopreservation. Following the cancer treatment regimen, the ovary can then be relocated into the pelvis via minimally invasive laparoscopic surgery for traditional IVF oocyte retrieval (32). Ovarian shielding and transposition might become a beneficial routine in the future, since the risk of miscarriage is not increased when ovaries were shielded (RR = 0.90, *p* = 0.88) (33). Alternatively, ovarian tissue cryopreservation and gonadal suppression using GnRHa remain an experimental option.

The standard FP options for post-pubertal males are sperm cryopreservation and gonadal shielding. The options for obtaining sperm cells are masturbation, TESE, or electroejaculation. Gonadal shielding, in this case for the testes, has been shown to reduce 3–10 fold reduction in radiation dose, which leads to <1% of the patient's prescript dose (34). The procedure may reduce the radiation dose to 0.5Gy in most radiotherapy treatments, which only leads to temporary oligospermia (35). This non-invasive procedure may be useful to be applied as a routine. In addition, nerve-sparing surgery as to not damage the nerves involved in ejaculation is also offered for patients in India (32).

India also offers third option parenting, allowing oocytes donation for infertile females. A 42-year-old woman with bilateral ductal carcinoma of the breast has been able to conceive with full term baby with no congenital anomalies by IVF performed with donor oocyte (36). The male counterpart of third-party sperm donation has never been reported, although this does not seem to be restricted by the Law (37).

FP in India are not covered by insurance. Fortunately, in some cases, tissue storage costs are, covered by in-house funding or grants (24).

## China

FP practice in China had been happening prior to the establishment of Chinese Fertility Preservation Society (CFPS) in 2017. CFPS was then founded with the aim of advancing research and clinical practice of FP, as well as enhance public awareness of FP especially oncofertility in China. However, CFPS President admits that there have not been any specific clinical regulations nor guidelines regarding FP in China, which may translate to overlooked FP options when cancer patients are treated. Opportunely, CFPS continues to move forward with its collaboration with international society, namely Oncofertility Professional Engagement Network (38). In addition, a survey among reproductive health professionals in China reveals a positive attitude toward interdisciplinary collaboration despite the lack of knowledge for standard (39). This study shows the continuous effort to provide information about the need and expectation for FP development in China.

In the research field, China has shown several novel findings for FP. The biotechnology advancement has allowed 3D *in vitro* follicle growth and organ-on-a-chip to be applied for ovarian tissue cryopreservation (38). Potentially, an ovary-on-a-chip will be able to further demonstrate the intricate reproductive physiological process as well as the potential toxicological effects of medical treatments (40). Also, Israel collaborates with China as the first to have achieved the initial step with regards to *in-vitro* maturation of spermatocytes. The chemotherapy (busulfan) treated spermatogonial cells shows proliferation and development into sperm-like cells using methylcellulose as a 3D *in-vitro* culture system (41). This will hopefully be a solution for FP in pre-pubertal male patients.

## Singapore

Singapore does not have a society for Fertility Preservation. However, the Singapore Hospital (SGH) and several private practices offer FP to the patients. SGH is the national referral center and the oldest and largest tertiary hospital in Singapore. The Center for Assisted Reproduction (CARE) by SGH is a one-stop center offering a full range of assessment services and proven assisted reproduction procedures, to help couples address infertility issues when starting a family including FP options. CARE provides Oocyte, sperm, embryo, and ovarian tissue cryopreservation including gamete, sperm, and embryo donation (42). SingHealth group offers an informative website available for the public regarding medical interventions including FP, but there is no specific section regarding the options (43).

SGH reported that in 2012 a method of FP through cryopreserved ovary has been successful. The 40-year-old woman patient had breast cancer thus the ovary was frozen for 3 years. Three months after the re-transplantation, menstruation resumes indicating resumed ovulation (44).

Singapore is one of the leading developers for novel FP methods. The first pregnancy and live birth resulting from cryopreserved embryos obtained from IVM oocytes after oophorectomy in an ovarian cancer patient achieved in 2013 in Singapore (45). In 2017, one experimental case for FP has also been reported on a pre-pubertal 13-year-old boy with beta-thalassemia major. Nine months following hCG and FSH administration to induce puberty and spermatogenesis, three adequate samples of sperm were obtained and cryopreserved (46). This is the first method that can be offered as an alternative since IVM of sperm from spermatogonial cell lines is not well-established.

With regards to funding, Singaporean government covers 35–75% of the cost of IVF procedure with the cost resides within a range of $6,000–$13,000 (47). However, FP in Singapore is restricted by the law such that it is only allowed based on medical reason (48).

## Indonesia

Indonesia has yet to establish the society for FP regardless of its ongoing practice. Initiatives to create FERTI-protect team in Indonesia has been sounded and is an on-going process. The referral system has also not been well-established. Nonetheless, human tissue from fertility centers can be referred to the FERTI-protect team for further applications. Information toward the patients is mostly managed by private practice that offers FP techniques. Regardless, the FP being offered in one of the centers are all well-established technique (49). It is generally agreed that multi-disciplinary team approach to FP is encouraged. Seminars regarding FP awareness for the experts and health practitioners have also been promoted by the Indonesian Obstetrics and Gynecology Association (POGI).

Researchers and clinicians have published several articles regarding FP in Indonesia. A study regarding the attitudes and knowledge of medical doctors specializing in obstetrics and gynecology found that there is a lack of knowledge about FP options, leading to sub-optimal provision of information to patients (50). Most participants only felt knowledgeable about pre-treatment with GnRH Agonists. The practitioners seem to be willing to provide FP with 92.5% agreed that FP is a high priority to discuss with newly diagnosed cancer patients, and 80% suggested FP for their patients, although 45% agreed that treating the primary cancer was more important than FP. With regards to referring the patients, only 35% reported having referred patients to a fertility specialist, and 15.1% provided patients with written information.

Among the patients with Turner Syndrome, fertility issue is more concerning than health and social issues. There seem to be a very high expectation for preserving fertility among these patients (85%) and they would like to undergo fertility treatment (97.5%) (51). This expectation, however, is challenged by their lack of information, fear of complication, and the cost of fertility treatment.

As a religious country, third party parenting is not allowed in Indonesia. The universal health coverage offered by the government does not include FP options and insurance companies also do not cover the cost. The main barriers to for offering discussions regarding FP in Indonesia were poor success rates (97.5%), affordability (93.8%), poor prognosis (92.6%), lack of obstetrician and gynecologists' knowledge (91.3%), and lack of fertility services in their area (81.3%) (50).

## Other Asian Countries

FP status in some Asian countries has been discussed (Table 1). Several other countries in Asia, for instance, Malaysia, Vietnam, and Iran just to name a few, face similar condition as in Indonesia. The practice of FP has been done by the experts although there is no society of FP in the country. As expected, a survey in Iran found that only 15% of all parents reported that they are aware of the danger of cancer treatment on fertility and only one-third of these patients received the knowledge from the treating physicians. Furthermore, the survey also found that despite a small percentage of success, many parents would still prefer to try FP for their sons (52). This situation that is surely detrimental for the patients' quality of life might also be applicable to other Asian countries.

**Table 1:** Summary of fertility preservation status in Asian countries.

**Country**	**Established FP society**	**Local guideline**	Information for patients^*^	**Referral system**	**Legal involvement**	**Insurance coverage**
Japan	Yes	Yes	Yes	Yes	Yes	Partial
Korea	Yes	Yes	Yes	No	No	Partial
India	Yes	No	Yes	No	No	No
China	Yes	No	No	No	Yes	No
Singapore	Yes	No	No	No	No	No
Indonesia	No	No	No	No	Yes	No

* *Considers the availability of official website that openly provides FP information for patients*.

Practitioners in these countries might also face legal and ethical challenges. With no guideline that addresses the legal and cultural aspect, FP practice is bound to face some issues. In Israel, where there is no direct policy in the issue, existing guidelines are often vague and ignored by the physicians. Furthermore, roughly half of the physicians are willing to perform more innovative procedures if backed by official guidelines (53). The lack of a support system for practitioners in several Asian countries will ultimately lead to poor conduct of FP and might be detrimental for the patients.

## Conclusion

FP is a highly demanded field and may improve the quality of life among patients. Further research should be conducted to explore new methods. Ideally, FP society should be established to promote a multi-disciplinary approach between practitioners, produce policies, and promote referral system at the very least for the benefit of the patients.

## Author Contributions

AH and VS write and prepare the manuscript. MM and BW supervised manuscript writing.

### Conflict of Interest Statement

The authors declare that the research was conducted in the absence of any commercial or financial relationships that could be construed as a potential conflict of interest.
